# Sequence polymorphism data of the hypervariable regions of mitochondrial DNA in the Yadav population of Haryana

**DOI:** 10.1016/j.dib.2018.03.004

**Published:** 2018-03-08

**Authors:** Kapil Verma, Sapna Sharma, Arun Sharma, Jyoti Dalal, Tapeshwar Bhardwaj

**Affiliations:** a Department of Genetics, Maharshi Dayanand University, Rohtak, Haryana 124001, India; b Govt. of Himachal Pradesh, Junga, Himachal Pradesh 173216, India

**Keywords:** Genetic variation, Hypervariable regions, mtDNA, Forensic, Yadav

## Abstract

Genetic variations among humans occur both within and among populations and range from single nucleotide changes to multiple-nucleotide variants. These multiple-nucleotide variants are useful for studying the relationships among individuals or various population groups. The study of human genetic variations can help scientists understand how different population groups are biologically related to one another. Sequence analysis of hypervariable regions of human mitochondrial DNA (mtDNA) has been successfully used for the genetic characterization of different population groups for forensic purposes. It is well established that different ethnic or population groups differ significantly in their mtDNA distributions. In the last decade, very little research has been conducted on mtDNA variations in the Indian population, although such data would be useful for elucidating the history of human population expansion across the world. Moreover, forensic studies on mtDNA variations in the Indian subcontinent are also scarce, particularly in the northern part of India. In this report, variations in the hypervariable regions of mtDNA were analyzed in the Yadav population of Haryana. Different molecular diversity indices were computed. Further, the obtained haplotypes were classified into different haplogroups and the phylogenetic relationship between different haplogroups was inferred.

**Specifications Table**

**Table:** 

Subject area	Forensic Science
More specific subject area	Forensic Genetics
Type of data	Tables and Figure
How data was acquired	Data was acquired by extracting, amplifying, sequencing and analysing the target region of mtDNA from the blood samples by using SureCycler 8800 (Agilent Technologies, USA), Gel Documentation System (Alpha Innotech, USA) DNA sequencer (Applied Biosystems by Life Technologies, CA, USA) Arlequin software version 3.5 (Computational and Molecular Population Genetics Lab, Zoological Institute, Switzerland), HaploGrep 2 software (Medical University of Innsbruck, Austria)
Data format	Analysed
Experimental factors	Blood sample collection, DNA Extraction, PCR Amplification, Sequencing and Interpretation of Data
Experimental features	During the experiments of extraction and amplification, the contamination is eliminated by using the filtered pipette tips, gloves, masks, lab coats, autoclaving of stock chemicals/tubes and separation of pre and post amplification areas in the laboratory
Data source location	Haryana (A northern state of India)
	Latitude: 29.0588°N
	Longitude: 76.0856°E
Data accessibility	The data is available with this article

**Value of the data**

• The present data is highly useful for the identification of individuals hypervariable involved in mass disasters, missing person cases and criminal cases in the Yadav population of Haryana.
• This data will help assess matches in mtDNA sequences in forensic casework in Haryana, and will be useful for population analyses based on specific sequence polymorphisms in the Yadav population of Haryana.
• The data report will provide baseline information for genetic studies based on the control region of mtDNA for tracking families related to the Yadav population of Haryana.
• This report is important for anthropological and evolutionary research, as well as for phylogenetic studies on the Yadav population of Haryana.
• This report could also be used by evolutionary biologists to study genetic variations in order to understand the possible relationships of the Yadav population with other populations.
• The data presented here can be used as reference material for future genetic studies on the Yadav population of Haryana.
• The mtDNA haplogroups generated in this data report can be used for tracing the migration and ancestry of the Yadav population of Haryana.
• The present data will contribute to the DNA database for the Yadav population of Haryana, which can be used for calculating the probability of matches based on mtDNA.

## 1 Data

**Fig. 1:**
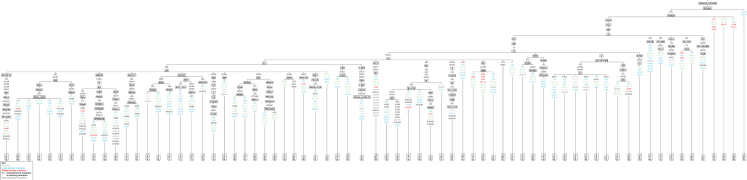
Phylogenetic tree of haplogroups including all related polymorphisms relative to the rCRS for Yadav population.

• Table 1: Primers used for amplification of the hypervariable regions (HVI, HVII and HVIII). Nucleotide position, primer name, sequence, length and melting temperature of the primers are mentioned.
• Table 2: Constituents of the PCR reaction mixture for each reaction volume of 25 µl per sample for amplification of the HVI, HVII and HVIII regions.
• Table 3: PCR cycling conditions for amplification of three hypervariable regions of mitochondrial DNA.
• Table 4: Molecular diversity indices for the HVI region, HVII+HVIII region & HVI+HVII+HVIII region.
• Table 5: Frequency distribution of the mtDNA haplotypes in the Yadav population.
• Table 6: Sequence polymorphism of the three hypervariable regions and their respective haplogroups in the Yadav population.
• Table S1: GenBank accession numbers for the mtDNA polymorphisms identified in the Yadav population.
• Figure 1: Phylogenetic tree of haplogroups including all related polymorphisms relative to the rCRS for Yadav population

## 2 Experimental design, materials and methods

### 2.1 Blood samples and DNA extraction

Blood samples were collected from 66 maternally unrelated individuals belonging to the Yadav population of Haryana from nearly all districts of Haryana. A sample of 2–5 ml of venous blood was drawn into 5 ml EDTA vacutainer tubes (Greiner Bio-One, USA). A consent form was signed by each participating individual at the site of collection. DNA was extracted using the phenol-chloroform-isoamyl (PCI) method [1].

### 2.2 PCR amplification

The three hypervariable regions were amplified by using two sets of PCR reactions. The primers F15900 and R00159 were used to amplify the HVI region. The primers F00015 and R00599 were used to amplify the HVII and HVIII regions [2] (Table 1). They were synthesized at Integrated DNA Technologies (IDT, USA). The PCR reaction was carried out in a final volume of 25 µl (Table 2). PCR was performed on SureCycler 8800 (Agilent Technologies, USA) (Table 3). Positive and negative controls were also used to ensure that no contamination was present at any stage during the experiments. The amplified PCR product was visualized using 1.6% agarose gel in the Gel Documentation System (Alpha Innotech). The amplified PCR product was cleaned with a GeneJET PCR Purification Kit (Thermo Fisher, USA) according to the manufacturer's guidelines to remove any impurities present in the template.

**Table 1:** Primers used for amplification of the hypervariable regions (HVI, HVII and HVIII). Nucleotide position, primer name, sequence, length and melting temperature of the primers are mentioned.

MtDNA region	Nucleotide position	Primers	Primer sequence (5′-3′)	Length (Bases)	Tm Value	PCR product Size (bp)
HVI	16024–16365	L15900 (F)	TACACCAGTCTTGTAAACC	19	49.1 °C	828
		H00159 (R)	AAATAATAGGATGAGGCAGGAATC	24	52.5 °C	
						
HVII & HVIII	73–576	L00015 (F)	CACCCTATTAACCACTCACG	20	52.7 °C	585
		H00599 (R)	TTGAGGAGGTAAGCTACATAA	21	50.3 °C

**Table 2:** Constituents of the PCR reaction mixture for each reaction volume of 25 µl per sample for amplification of the HVI, HVII and HVIII regions.

**S.No.**	**Chemical**	**Quantity**
1	10 × PCR buffer	2.5 µl
2	0.2 mM of each dNTPs	2.5 µl
3	0.6 μM each of forward primer (F)	1.5 µl
4	0.6 μM each of reverse primer (R)	1.5 µl
5	5 Units/ µl of Taq DNA polymerase	0.5 µl
6	D/DH_2_O	15.5 µl
7	50 ng of template DNA	1 µl

**Table 3:** PCR cycling conditions for amplification of three hypervariable regions of mitochondrial DNA.

**Cycle Step**	**Temperature**	**Time duration**
Hot start	95 °C	11 min
Denaturation	95 °C	15 s
Annealing	62 °C	30 s
Elongation	72 °C	1 min
End cycle Elongation	72 °C	10 s
Hold	4 °C	∞

### 2.3 Sequencing

The cleaned PCR product was sequenced by commercial DNA sequencing service (Xcelris Labs Limited, Ahmedabad, India). Both the strands were sequenced with the ABI BigDye Terminator Cycle Sequencing Kit on the ABI 3700 Genetic Analyzer (Applied Biosystems). All the samples were sequenced with the same primers used in PCR amplification of the HVI, HVII and HVIII regions. An additional primer (16410R-GAGGATGGTGGTGGTCAA) was used in cases where there was slippage due to ‘C’ stretch in hyper variable region 1.

### 2.4 Statistical analysis

A total of 66 mtDNA sequences was obtained, which included all the base pairs from nucleotide positions 16021−16365, 69−576 bp. Sequence data files were edited and aligned with the revised Cambridge reference sequences (rCRS) [3] by using the Mega 7 software [4]. Manual alignment was also done to cross check the results by creating data analysis sheets. The interpretation was done as per the guidelines [5], [6], [7]. The coding for heteroplasmic sites was done according to the IUPAC codes in the interpretation guidelines to interpret the mtDNA data analysis results [8]. Any observed C-stretch length heteroplasmy in the HVI, HVII and HVIII region sequences was excluded from statistical analysis. Statistical analysis was first performed for each hypervariable segment separately, and then combined for the HVI+HVII+HVIII regions. Gene diversity was calculated according to Tajima [9]. Population pairwise differences were determined based on genetic distances [10]. Haplotype diversity, mean pairwise differences, nucleotide diversity, Harpending's raggedness index, mismatch distributions, Fu's Fs and Tajima's D test statistics were calculated using the Arlequin software, version 3.5.1.2 [11] (Table 4). A Random match probability (RMP) was calculated according to Stoneking et al. [12] (Table 5). Haplogroup classification was performed using the HaploGrep 2 software [13] (Table 6). A phylogenetic tree of all the haplogroups was constructed using HaploGrep 2, while all the classified samples were combined to produce a resulting (rooted) tree, which included all the related polymorphisms relative to the rCRS (Fig. 1). GenBank accession numbers for the mtDNA polymorphisms identified in the Yadav population are provided in Table S1 (Supplementary table).

**Table 4:** Molecular diversity indices for the HVI region, HVII+HVIII region & HVI+HVII+HVIII region.

**Diversity indices**	**HVI**^a^**region**	**HVII**^b^**+HVIII region**	**HVI+HVII+HVIII**^c^**region**
No. of polymorphic sites	50	55	105
No. of observed transitions	48	41	89
No. of observed transversions	4	10	14
No. of observed substitutions	52	51	103
No. of observed indels	–	5	5
Nucleotide composition (%) C	33.31	34.59	34.07
T	22.41	22.76	22.62
A	33.01	30.45	31.49
G	11.27	12.20	11.82
Mean number of pairwise differences	4.27 ± 2.14	5.5603 ± 2.7054	9.8331 ± 4.5583
Heterozygosity/sample	0.086 ± 0.09	0.1013 ± 0.12	0.09433 ± 0.11
No of haplotypes	50	55	66
Gene diversity	0.9869 ± 0.0065	0.9925 ± 0.0046	1.000 ± 0.0026
Nucleotide diversity	0.01238 ± 0.0068	0.01092 ± 0.0058	0.01151 ± 0.005918
Ss2 of haplotype frequencies (RMP)	0.028	0.0225	0.0152
Alleles frequency (Mean ± S.D)	1.151 ± 0.374	1.11 ± 0.31	1.156 ± 0.388
Sum of square deviation	0.0045	0.0028	–
Harpending's raggedness index	0.0159	0.0103	–
Mismatch distribution observed mean	4.273	5.560	–
Mismatch observed variance	4.62	6.031	–
Tajima's D test	−1.9763	−1.8781	−1.9896
Fu's FS test	−25.8062	−25.3770	−24.6038

a Hypervariable region I.

b Hypervariable region II.

c Hypervariable region III.

**Table 5:** Frequency distribution of the mtDNA haplotypes in the Yadav population.

**Number of times a haplotype repeated**	**Numbers of Haplotypes**		
	**HVI region**	**HVII + HVIII region**	**HVI + HVII + HVIII region**
1	40	48	66
2	7	4	–
3	2	2	–
4	–	1	–
6	1	–	–
Total	50	55	66
**Random Match Probability**	0.028	0.022	0.015

**Table 6:** Sequence polymorphism of the three hypervariable regions and their respective haplogroups in the Yadav population.

**Sample ID**	**HVI region**	**HVII region**	**HVIII region**	**Haplogroup**
YA1	16129A 16242T 16356C	263G 309.1CC 315.1C		H1b
YA2	16189C 16223T 16278T 16352C 16362C	73G 151T 152C 263G 315.1C 389R	523d 524d	L3b1a+@16124
YA3	16095T 16223T 16335G	73G 263G 309.1C 315.1C	489C	M
YA4	16292T	73G 146C 150T 189G 194T 195C		W5a2
		204C 207A 212K 263G 309.1C 315.1C		
YA5	16111A 16223T	73G 195C 263G 309.1C 315.1C	482C 489C 523d 524d	M21b+210
YA6	16036R 16189C 16223T 16234T 16311C	73G 146C 152C 234G 263G 309.1C 315.1C		N9b
YA7	16309G 16318T	73G 204C 217C 263G 315.1C	482C 489C	U7
YA8	16192T 16223T 16278T	73G 152C 222S 263G 309.1C 315.1C	489C	M73'79
YA9	16309G 16318T	73G 195A 204C 207A 263G 309.1C 315.1C	489C 523d 524d	M30g
YA10	16126C 16181G 16209C 16362C	73G 194T 263G 309.1C 315.1C	489C 523d 524d	D4b2b2b
YA11	16223T 16318T 16325C	73G 135G 195C 263G 309.1C 315.1C	573.1C	N
YA12	16048A 16129A 16223T	73G 152C 204Y 214G 263G 315.1C	461T 489C 523d 524d 573.1C	M5b2
YA13	16126C 16223T 16311C	195C 263G 309.1C 315.1C	499A	H11
YA14	16309G 16318C	73G 143A 189G 194T 195C 204C 207A 263G 309.1C 315.1C		W+194
YA15	16174T 16354T	73G 195C 204C 263G 309.1C 315.1C	482C 489C	M3a1+204
YA16	16126C 16223T 16311C	73G 152C 263G 309.1C 315.1C		L3h1
YA17	16111T 16184T 16189C 16223T 16274A 16295T	150T 263G 309.1CC 315.1C		M37e
YA18	16036R 16048A 16129A 16218T 16223T	154C 195C 235G 263G 309.1C 315.1C	456T 480C	H5'36
YA19	16150T 16223T 16298C 16327T 16357C	73G 114T 146C 263G 309.1C 315.1C	489C	C4a2
YA20	16036R 16309G 16318T	73G 154C 195C 235G 263G 309.1C 315.1C		U7
YA21	16126C 16129A 16183C 16304C	73G 146C 152C 195A 263G 315.1C	489C 523d 524d	M30c
YA22	16093W 16172C 16293G 16304C 16362C	73G 152C 263G 309.1C 315.1C	489C 524.1AC	D4b2b2b
YA23	16095T 16184T 16223T 16249C 16359C	73G 152C 263G 309.1C 315.1C	523d 524d	M34a1
YA24	16111T 16183C 16189C 16192T 16223T 16292T 16311C 16325C 16355T	73G 146C 189G 195C 207A 263G 315.1C		W6
YA25	16036R 16355T	73G 186G 204C 217C 263G 309.1C 315.1C	489C 524.1AC	D4a6
YA26	16111Y 16129R 16189Y 16218Y 16223T 16293Y 16311Y	73G 151T 152C 263G 309.1C 315.1C	544d	N5
YA27		253T 263G 309.1C 315.1C	478T 544d	H2a2a
YA28	16266T 16304C	73G 174Y 204C 217C 263G 315.1C	444M 482C 489C	M3a1+204
YA29	16183C 16189C 16193.1C	73G 263G 309.1C 315.1C	443M 489C	M1a3b1
YA30	16036R	73G 151T 152C 263G 315.1C	443M	U8c
YA31	16095T 16184T 16223T 16249C 16359C	73G 249d 263G 315.1C	489C	M34a1
YA32		73G 152C 162G 194T 198A 204C 263G 315.1C	523d 524d	H32
YA33	16189C 16223T	73G 239d 240C 243C 245.1A 263G 309.1C 315.1C	523d 524d	N9b
YA34	16223T	73G 93G 200G 263G 309.1C 315.1C	523d 524d	N
YA35	16309G 16318T	73G 146C 263G 309.1C 315.1C	489C	U7
YA36	16126C 16186T 16223T	73G 143A 189G 194T 195C 204C 207A 263G 309.1C 315.1C		W+194
YA37	16126C 16362C	263G 309.1CC 315.1C		H14b1
YA38	16309G 16318T 16343R 16362Y	73G 204C 263G 315.1C	489C	U7
YA39	16223T 16263C	73G 263G 309.1C 315.1C	489C	M50
YA40	16223T 16292T	73G 150T 263G 309.1C 315.1C 373G		R30b2
YA41	16126C 16154C 16223T 16224C	73G 263G 315.1C	489C 524.1AC	M3c2
YA42	16126C 16129A 16242T	73G 263G 315.1C	489C	JT
YA43	16126C 16223T 16311C	73G 151T 152C 263G 309.1C 315.1C	523d 524d	L3h1
YA44	16129A 16223T 16265C	73G 152C 195A 263G 309.1C 315.1C	489C 523d 524d	M5a2a1a
YA45	16145A 16223T	73G 153G 195C 225A 226C 263G 315.1C		X2b+226
YA46	16036R 16048A 16129A 16218T 16223T	73G 151T 152C 263G 315.1C	523d 524d	M5b2
YA47		73G 146C 246C 263G 315.1C	489C	M18'38
YA48	16036R 16223T 16362C	73G 204C 263G 309.1C 315.1C	489C 523d 524d	M9
YA49	16036R 16193T 16309G 16318T	73G 152C 263G 309.1CC 315.1C	523d 524d	U7
YA50	16126C 16181G 16209C 16362C	73G 152C 263G 315.1C		R30a1b
YA51	16126C 16192T 16223T 16312G	73G 152C 263G 271T 295T 315.1C	459d 462T 489C 523d 524d	M37+152
YA52	16036R 16183C 16189C 16193.1C	73G 151T 152C 263G 315.1C		U8c
YA53		263G 309.1C 315.1C	544.1C	H2a2a
YA54	16036R 16223T	73G 195A 204C 263G 315.1C	489C 523d 524d	M30g
YA55	16126C 16192T 16223T 16312G	73G 128T 194T 195C 204C 263G 315.1C	482C 489C	M3a1+204
YA56	16036R 16126C 16223T 16324C	73G 204C 263G 315.1C	482C 489C	M3a1+204
YA57	16126C 16223T 16266T 16311C	73G 204C 217C 263G 315.1C	482C 489C	M3a1+204
YA58	16309G 16318T 16343G 16362C	73G 153G 263G 309.1C 315.1C	463T 485C 489C	U7
YA59	16036R	73G 152C 263G 309.1C 315.1C 373G		R30b
YA60		263G 315.1C	467T 476A 544.1C	H2a2a
YA61	16036R 16223T 16292T 16311C	73G 189G 195C 204C 207A 263G 315.1C		W
YA62		73G 146C 152C 263G 309.1C 315.1C	523d 524d	H32
YA63	16174Y 16223Y 16243Y 16304Y	73G 151T 152C 263G 315.1C	523d 524d	D4h1c
YA65	16129A 16223T	73G 150T 214G 263G 309.1C 315.1C 333C	489C 575T	M5c1
YA66	16129A 16223T 16265C	263G 309.1C 315.1C	489C	M5a2a1a
YA70	16189C 16223T	73G 152C 158K 263G 309.1C 315.1C	523d 524d	N9b

## Figures and Tables

**Fig. S1:**
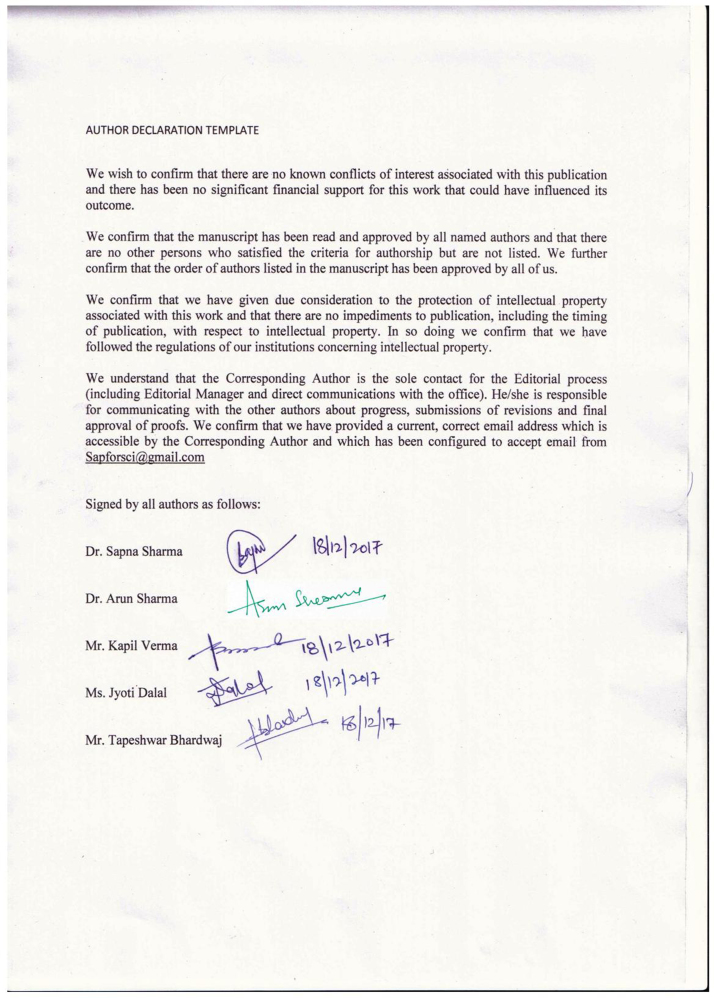


## Footnotes

Appendix A Supplementary data associated with this article can be found in the online version at 10.1016/j.dib.2018.03.004.
