# Supplementary material for: Sequence polymorphism data of the hypervariable regions of mitochondrial DNA in the Yadav population of Haryana
Source: Data Brief. 2018 Mar 8;18:164–71. doi: 10.1016/j.dib.2018.03.004 (PMC5996146; doi:10.1016/j.dib.2018.03.004)
Supplement: Supplementary file 1 — Supplementary material [file mmc2.docx]

**Supplementary table:**

**Table 7**: GenBank accession numbers for the mtDNA polymorphisms identified in the Yadav population

| ID Code | Accession Number | ID Code | Accession Number | ID Code | Accession Number |
| --- | --- | --- | --- | --- | --- |
| YA1 | MG456610 | YA23 | MG456632 | YA45 | MG456654 |
| YA2 | MG456611 | YA24 | MG456633 | YA46 | MG456655 |
| YA3 | MG456612 | YA25 | MG456634 | YA47 | MG456656 |
| YA4 | MG456613 | YA26 | MG456635 | YA48 | MG456657 |
| YA5 | MG456614 | YA27 | MG456636 | YA49 | MG456658 |
| YA6 | MG456615 | YA28 | MG456637 | YA50 | MG456659 |
| YA7 | MG456616 | YA29 | MG456638 | YA51 | MG456660 |
| YA8 | MG456617 | YA30 | MG456639 | YA52 | MG456661 |
| YA9 | MG456618 | YA31 | MG456640 | YA53 | MG456662 |
| YA10 | MG456619 | YA32 | MG456641 | YA54 | MG456663 |
| YA11 | MG456620 | YA33 | MG456642 | YA55 | MG456664 |
| YA12 | MG456621 | YA34 | MG456643 | YA56 | MG456665 |
| YA13 | MG456622 | YA35 | MG456644 | YA57 | MG456666 |
| YA14 | MG456623 | YA36 | MG456645 | YA58 | MG456667 |
| YA15 | MG456624 | YA37 | MG456646 | YA59 | MG456668 |
| YA16 | MG456625 | YA38 | MG456647 | YA60 | MG456669 |
| YA17 | MG456626 | YA39 | MG456648 | YA61 | MG456670 |
| YA18 | MG456627 | YA40 | MG456649 | YA62 | MG456671 |
| YA19 | MG456628 | YA41 | MG456650 | YA63 | MG456672 |
| YA20 | MG456629 | YA42 | MG456651 | YA65 | MG456673 |
| YA21 | MG456630 | YA43 | MG456652 | YA66 | MG456674 |
| YA22 | MG456631 | YA44 | MG456653 | YA70 | MG456675 |
